# Altered collagen I and premature pulmonary embryonic differentiation in patients with OI type II

**DOI:** 10.14814/phy2.15737

**Published:** 2023-07-04

**Authors:** S. Storoni, L. Celli, M. Breur, D. Micha, S. J. E. Verdonk, A. Maugeri, J. G. van den Aardweg, M. Riminucci, E. M. W. Eekhoff, M. Bugiani

**Affiliations:** ^1^ Department of Internal Medicine Section Endocrinology Amsterdam UMC Location Vrije Universiteit Amsterdam Amsterdam The Netherlands; ^2^ Amsterdam Movement Sciences Amsterdam The Netherlands; ^3^ Department of Pathology Amsterdam University Medical Centre Amsterdam The Netherlands; ^4^ Department of Human Genetics Amsterdam UMC Location Vrije Universiteit Amsterdam Amsterdam The Netherlands; ^5^ Department of Respiratory Medicine Amsterdam University Medical Center Amsterdam The Netherlands; ^6^ Department of Molecular Medicine Sapienza University Rome Italy

**Keywords:** biochemical regulator, cell differentiation, collagen type I, lung development, osteogenesis imperfecta

## Abstract

Pulmonary hypoplasia and respiratory failure are primary causes of death in patients with osteogenesis imperfecta (OI) type II. OI is a genetic skeletal disorder caused by pathogenic variants in genes encoding collagen type I. It is still unknown if the collagen defect also affects lung development and structure, causing lung hypoplasia in OI type II. The aim of this study was to investigate the intrinsic characteristics of OI embryonic lung parenchyma and to determine whether altered collagen type I may compromise airway development and lung structure. Lung tissue from nine fetuses with OI type II and six control fetuses, matched by gestational age, was analyzed for TTF‐1 and collagen type I expression by immunohistochemistry, to evaluate the state of lung development and amount of collagen. The differentiation of epithelium into type 2 pneumocytes during embryonic development was premature in OI type II fetuses compared to controls (p < 0.05). Collagen type I showed no significant differences between the two groups. However, the amount of alpha2(I) chains was higher in fetuses with OI and the ratio of alpha1(I) to alpha2(I) lower in OI compared to controls. Cell differentiation during lung embryonic development in patients with OI type II is premature and impaired. This may be the underlying cause of pulmonary hypoplasia. Altered cell differentiation can be secondary to mechanical chest factors or a consequence of disrupted type I collagen synthesis. Our findings suggest that collagen type I is a biochemical regulator of pulmonary cell differentiation, influencing lung development.

## 1 INTRODUCTION

Acute and chronic respiratory symptoms are responsible for decreased quality of life and are a major cause of death in patients with osteogenesis imperfecta (OI) (Folkestad, 2018; Folkestad et al., 2016; McAllion & Paterson, 1996; Yonko et al., 2020). OI is a syndromic genetic disease of extreme bone fragility caused by molecular defects in collagen type I (Marini et al., 2017). Collagen type I is found in bone and other connective tissues, including the interstitial parenchyma of the mammalian lung (Bienkowski & Gotkin, 1995). Clinically, OI is characterized by bone fragility, small stature, skeletal deformities, ligament laxity, blue sclerae, dentinogenesis imperfecta, hearing impairment, and cardiopulmonary disease. According to the Sillence classification, OI type I is the least severe form, which usually presents with an insufficient level of collagen synthesis; these patients have usually no or very limited skeletal deformities (Maioli et al., 2019). OI types II, III, and IV are mainly characterized by structural alterations in type I collagen, leading to progressive bone deformation, multiple fractures, very short stature and wheelchair dependency (Forlino & Marini, 2016; Marini et al., 2017; Monti et al., 2010; Sillence et al., 1979; van Dijk et al., 2011; Van Dijk & Sillence, 2014). OI type II is the most severe, with mortality generally before birth. OI type II is associated with lung hypoplasia (Barros et al., 2016; Shapiro et al., 1989; Sillence et al., 1984; Thibeault et al., 1995). If patients survive birth, they commonly die in the early neonatal period due to pulmonary insufficiency (Ayadi et al., 2015; Himakhun et al., 2012). Other manifestations of OI type II include fragile and poorly ossified bones, limb growth failure, abnormal development of the rib cage, and decreased intrathoracic volume (Cole & Dalgleish, 1995; Sillence et al., 1984). The majority of perinatal lethal OI is due to autosomal dominant variants in *COL1A1* and *COL1A2* (Bodian et al., 2009). In addition to variants in collagen genes, lethal OI can also be caused by several autosomal recessive genes, such as *PPIB*, some of which have a direct role in collagen posttranslational modifications (Claeys et al., 2021).

In the last few years, attention to respiratory issues in OI has been gradually growing (Storoni et al., 2021). Yet, little is known regarding the underlying pathophysiology of decreased pulmonary function. Several studies have reported that altered respiratory function in OI is related to the severity of the disease and the anatomic configuration of the sternum, rib cage, or vertebral deformities (Falvo et al., 1973; Kaplan et al., 2013; LoMauro et al., 2012, 2018; Pan et al., 2006; Sanchis‐Gimeno et al., 2020; Wekre et al., 2014; Widmann et al., 1999). By contrast, other studies support the hypothesis that impaired lung function is intrinsic to OI, implying that altered type I collagen crucially impacts on the interstitial lung parenchyma, leading to restrictive or obstructive disease (Bronheim et al., 2019; Himakhun et al., 2012; Khan et al., 2020; Morikawa et al., 2016; Shapiro et al., 1989; Thibeault et al., 1995; Thiele et al., 2012). The most recent systematic review on this topic concluded that reduced lung function may deteriorate in relation to rib cage or vertebral deformities, but this is likely not the only cause of pulmonary disease in OI. Thus, collagen type I defects may still play a role in the development of the OI lung parenchyma, ultimately leading to a lung disorder (Storoni et al., 2021).

To date, the amount of type I collagen in lung tissue and its impact on lung development is incompletely elucidated. Type I collagen is widely distributed in the interstitium of the bronchial tree and primarily contributes to lung wall strength providing mechanical stability (Konomi et al., 1981; Liu et al., 2021). How and if collagen interacts with epithelial cells is still unclear. Respiratory epithelial cells are type 1 and 2 pneumocytes. Type 1 pneumocytes overlie the capillaries. These constitute the major gas exchange surface of the alveolus and contribute to the preservation of the permeability barrier function of the alveolar membrane. Type 2 pneumocytes are the precursors of type 1 pneumocytes, responsible for surfactant production and homeostasis. In some studies, important reciprocal interactions were found in co‐cultures of type 2 pneumocytes and fibroblasts, with the latter having a trophic effect on pneumocytes (Adamson et al., 1989; Griffin et al., 1993). Another study found that a reduction of the collagenous components can alter the functional state of type 2 pneumocytes, resulting in reduced surfactant synthesis (King & Adamson, 1987). As suggested by Sugihara et al., it would appear that differentiation of type 2 pneumocytes is supported by the collagen matrix (Sugihara et al., 1993).

Despite the aforementioned findings, knowledge on the influence of the extracellular matrix (ECM) and its modifications on epithelial function in the lungs is still inadequate. Our study aims at addressing this gap by investigating the intrinsic characteristics of OI lung parenchyma in OI fetal lung compared to control tissue. In addition, the study aims at determining whether altered collagen type I influences the development and physiological structure of the lung. To this aim, fetal lung tissue was analyzed for expression of thyroid transcription factor‐1 (TTF‐1), type 1 collagen, alpha1(I), and alpha2(I) by immunohistochemistry. TTF‐1 regulates lung morphogenesis and is most abundant in type 2 pneumocytes, where it controls surfactant protein synthesis; thus, its expression can be used as indicator of the lung developmental stage (DeFelice et al., 2003; Lazzaro et al., 1991; Zhou et al., 1996). This is the first study to examine the intrinsic characteristics of lung parenchyma in OI type II fetuses, shedding light on both the cell differentiation status and the potential role of collagen as a possible pathophysiological cause of lung hypoplasia in OI II.

## 2 MATERIALS AND METHODS

Formalin‐fixed paraffin‐embedded (FFPE) lung tissue of fetuses between the pseudoglandular (Week 5–17 gestational age [GA]) and canalicular development phase (Week 17–26 GA) was retrieved from the Department of Pathology of the Amsterdam UMC. Lung tissue from nine OI fetuses was compared to that of six GA‐matched fetuses without OI. Consent from the parents was secured in accordance with the guidelines of the Amsterdam Medical Centrum Ethical committee.

Lungs had been obtained at autopsy from fetuses suspected of OI on ultrasound. Genetic analysis confirmed the clinical OI diagnosis. Lungs from GA‐matched controls had been obtained from terminated pregnancies because of alterations unrelated to a connective tissue or lung disorder. Histopathology of control fetal lungs showed a histoarchitecture compatible with the GA and no pathological features. Table 1 illustrates the clinical data of the OI cases and controls. Two age categories were used for analysis: early GAs including fetuses from 14 to 18 weeks and later GAs including fetuses from 19 to 23 weeks.

**TABLE 1:** Baseline characteristics of studied fetuses.

Anonymized code	Gender	Gestational age (week + days)	Diagnosis	Cause of death	DNA analysis (HGVS nomenclature)	Lung macroscopy
fetus 001	M	14 + 5	OI II	TOP	COL1A1 c.1543G>C p.(Gly515Arg) (heterozygous) (ref. seq. NM_000088.3)	Normal*
fetus 006	M	15 + 5	OI II	TOP	COL1A1 c.1921G>A p.(Gly641Arg) (heterozygous) (ref. seq. NM_000088.3)	Normal
fetus 005	M	16 + 0	OI II	TOP	PPIB c.556_559del p.(Lys186Glnfs*8) (homozygous)	Normal
fetus 002	V	16 + 1	OI II	TOP	COL1A1 c.2893G>A p.(Gly965Ser) (heterozygous) (ref. seq. NM_000088.3)	Normal
fetus 010	M	18 + 0	OI II	TOP	COL1A1 c.1273G>A p.(Gly425Ser) (heterozygous) (ref. seq. NM_000088.3)	Normal
fetus 004	M	21 + 0	OI II	TOP	COL1A1 c.3830A>T (p.Asp1277Val) (heterozygous) (ref. seq. NM_000088.3)	Normal
fetus 003	V	22 + 1	OI II	TOP	Not identified	Normal
fetus 007	V	22 + 4	OI II	TOP	COL1A2 c.1856G>A p.(Gly619Glu) (heterozygous) (ref. seq. NM_000089.3)	Normal
fetus 008	V	22 + 4	OI II	TOP	COL1A1 c.1760G>A p.(Gly587Asp) (heterozygous) (ref. seq. NM_000088.3)	Lung hypoplasia

Anonymized code	Gender	Gestational Age (week+days)	Diagnosis		DNA analysis	Lung macroscopy
fetus control 3	M	14 + 3	Mega‐bladder	TOP	Array and QF‐PCR normal	Normal
fetus control 5	V	16 + 1	Cerebellar hypoplasia	TOP	Array and QF‐PCR normal	Normal
fetus control 2	V	18 + 1	Cervical insufficiency	Spontaneous	Array and QF‐PCR normal	Normal
fetus control 1	M	21 + 4	Fallot tetralogy	TOP	Array and QF‐PCR normal	Normal
fetus control 8	V	22 + 0	Aortic and mitral valve stenosis, coartatio, persistent vena cava superior sinistra	TOP	Array and QF‐PCR normal	Bilaterally two lobes
fetus control 10	M	23 + 5	Destruction defect of the right leg	TOP	Array and QF‐PCR normal	Normal

Abbreviation: TOP, termination of pregnancy.

* Normal: Histology, Lung (Khan & Lynch, 2022).

### 2.1 Histochemistry and immunohistochemistry

FFPE tissue was cut in 5 μm‐thick sections and routinely stained with hematoxylin and eosin (HE) and Elastic van Gieson (EvG). Immunohistochemical staining was carried out by rehydrating sections in xylene and alcohol. Endogenous peroxidase activity was blocked in 0.3% (w/v) H_2_O_2_ in PBS for 30 min, followed by heat‐induced antigen retrieval in citrate buffer (pH = 6) or TRIS/EDTA (pH = 9). Primary antibodies against thyroid transcription factor 1 (TTF‐1, 1:800, Dako, M3575), type I collagen (COL1, 1:20, Usbio, C7510‐17K), collagen type I alpha 1 chain (COL1A1, 1:100, Abnova, PAB17205), and collagen type I alpha 2 chain (COL1A2, 1:100, Sigma, SAB2100463) were incubated overnight at room temperature. The next day, slides were rinsed and incubated with horseradish peroxidase‐labeled secondary antibodies (EnVision Detection Systems HRP Rabbit/Mouse (DAKO, K5007), Rabbit Anti‐Goat HRP (DAKO, P0160)) and developed using 3,3′‐diaminobenzidine (DAB, 1:50, DAKO) for 10 min. Sections were counterstained with hematoxylin, dehydrated with alcohol and xylene, and mounted with Quick‐D (Klinipath, 7280). Histochemistry and immunohistochemical staining were performed on lung tissue from all nine OI fetuses and six control fetuses.

### 2.2 Digital pathology slide scanners

Light microscopy pictures were taken with a Leica DM6000B microscope (Leica microsystems). Pictures were acquired at 200× final magnification using “ViewScanner” with 600 ppi resolution, width 2592 pixels (2592 × 2076 × 24 BPP). For each fetus, ten 200× pictures were randomly acquired, five in the periphery of the lung and five centrally.

### 2.3 Analysis and statistics

All respiratory ducts from all pictures were counted. Five transversally cut respiratory ducts were selected in each picture to minimize random cutting errors.

**FIGURE 1:**
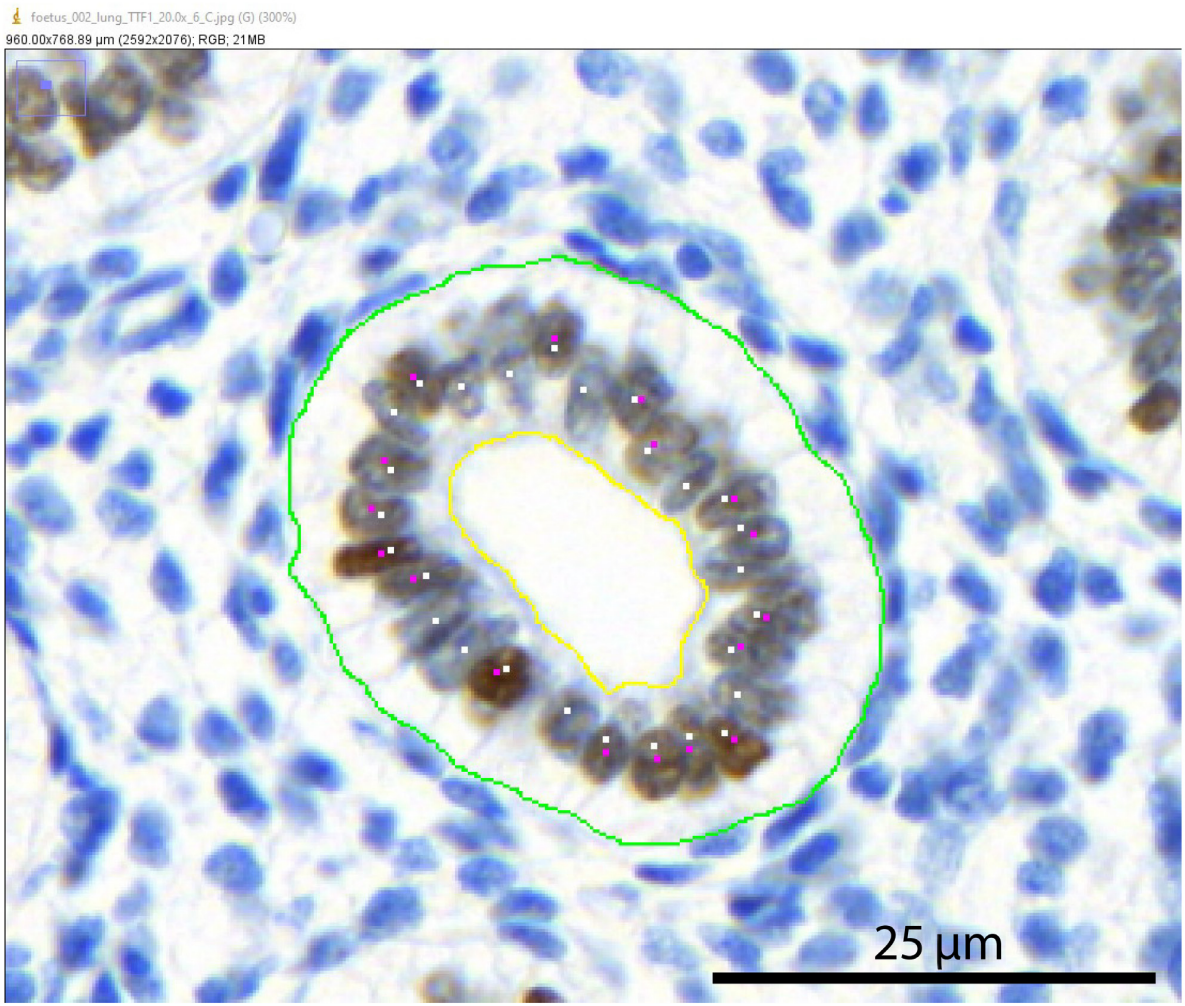
Microscopy picture of a respiratory duct. Using the “freehand selection” plugin of Image J, the area within each respiratory lumen was calculated, taking the apical membrane of the cells as a reference point (yellow line). The perimeter of the basal cell membrane was also measured (green line). The total number of cells per respiratory duct and number of TTF1‐positive cells were counted (white and pink dots). Scale bar is 25 μm.

For each fetus, 50 respiratory ducts were analyzed. ImageJ was used to analyze the pictures (Set scale: distance in pixels 540, known distance 200, pixel aspect ratio 1.0, unit of length μm, scale 2.7 pixels/um). Using the “freehand selection” plugin, the area within each respiratory lumen was calculated, taking the apical membrane of the cells as a reference point (yellow line in Figure 1). The perimeter of the basal cell membrane was also measured (green line in Figure 1). The total number of cells per respiratory duct and number of TTF1‐positive cells were counted (white and pink dots in Figure 1). The ratio of TTF1‐positive to total cells per 200 um respiratory duct basal membrane was calculated. These parameters were used to assess and compare respiratory duct development at different GAs.

Statistical analysis was performed using GraphPad Prism v9.3.1. Data are displayed as mean ± standard deviation (SD). Distribution of the data was determined using a Shapiro–Wilk test. Either a one‐ and two‐way ANOVA, an unpaired *t*‐test or a nonparametric Mann–Whitney *U*‐test was performed to evaluate the differences between OI and control fetuses. Differences were considered significant if *p* < 0.05.

## 3 RESULTS

Table 1 shows the gestational age, clinical diagnosis, and results of genetic analysis.

### 3.1 Lung parenchyma structure

Between Weeks 14 and 23 of GA, no macroscopic differences were found in lung lobulation between the OI and control group (Table 1) (Khan & Lynch, 2022). Only OI fetus 008 (22 + 4 weeks GA) showed, on macroscopic evaluation, pulmonary hypoplasia. Microscopy showed no relevant differences in lung histoarchitecture between the OI and control group. The average number of conducting airways and the number of terminal bronchioles were similar in both groups (Table S1 and Figure S1). There was no significant difference in the average area of airway ducts in the two groups. There were no significant differences in the average number of cells per airway duct and the length of the basal membrane between OI and control fetuses (Figure S1).

### 3.2 TTF‐1 expression

**FIGURE 2:**
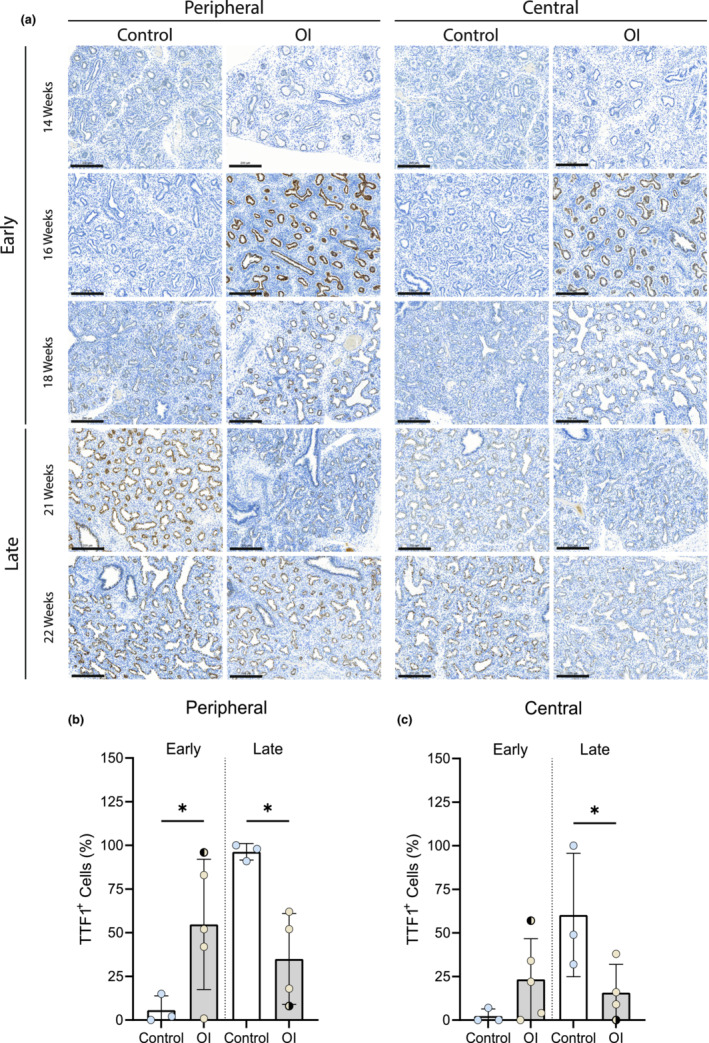
TTF‐1 expression in OI and control fetuses. (a) TTF‐1 expression in lungs from OI and control fetuses was detected in the nuclei of type 2 pneumocytes lining the bronchioles and respiratory structures by immunostaining. (b) Percentage of TTF‐1‐positive cells in the peripheral parenchyma of the early and late GA groups. (c) Percentage of TTF‐1‐positive cells in the central parenchyma of the early and late GA groups. *p < 0.05. Scale bar is 200 μm. 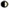: fetus 005 (*PPIB*). 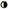: fetus 007 (*COL1A2*). The one‐way ANOVA statistical test was used. Error bars indicate the standard deviation (SD).

TTF‐1 expression regulates lung histomorphogenesis and it is most abundant in type 2 pneumocytes; its expression was used as indicator of the lung developmental stage. The proportion of TTF‐1 positive cells on the total cells per airway duct was greater at an earlier stage in the OI group compared to controls (Figure 2). At Week 14 GA, in both groups 0% of the total cells per circumference was positive for TTF‐1. At Week 16, all three OI fetuses demonstrated a high increase in TTF‐1 expression, with 52%, 77%, and 28% of positivity, respectively, of the total epithelial cells per circumference. In the control fetuses of Week 16 GA, there was still no positivity, and TTF1 expression started at Week 18 (Table S1). Only one OI fetus at Week 21 was available, in which TTF1 expression was 13%. At Week 22, both groups had a high expression of TTF1; three OI fetuses had 39%, 60%, and 45% of TTF1 expression, respectively, whereas the control fetuses had 70% and 100% of expression (Table S1).

When categorized into two groups, before and after 18 weeks of GA, a significant difference (calculated with one‐way ANOVA) was observed. Lung tissue of OI fetuses before Week 18 had higher and earlier expression of TTF‐1 compared to lung tissue of control fetuses, in whom TTF‐1 expression started later (Figure 2b,c). In the peripheral part of the lung, both during the early and late stage, a significant difference was seen between OI and control group (p:0,0281 and p:0,0231, respectively). In the central part of the lung during the late stage, a significant difference was found between OI and controls (p:0,0477).

### 3.3 Collagen type I expression

**FIGURE 3:**
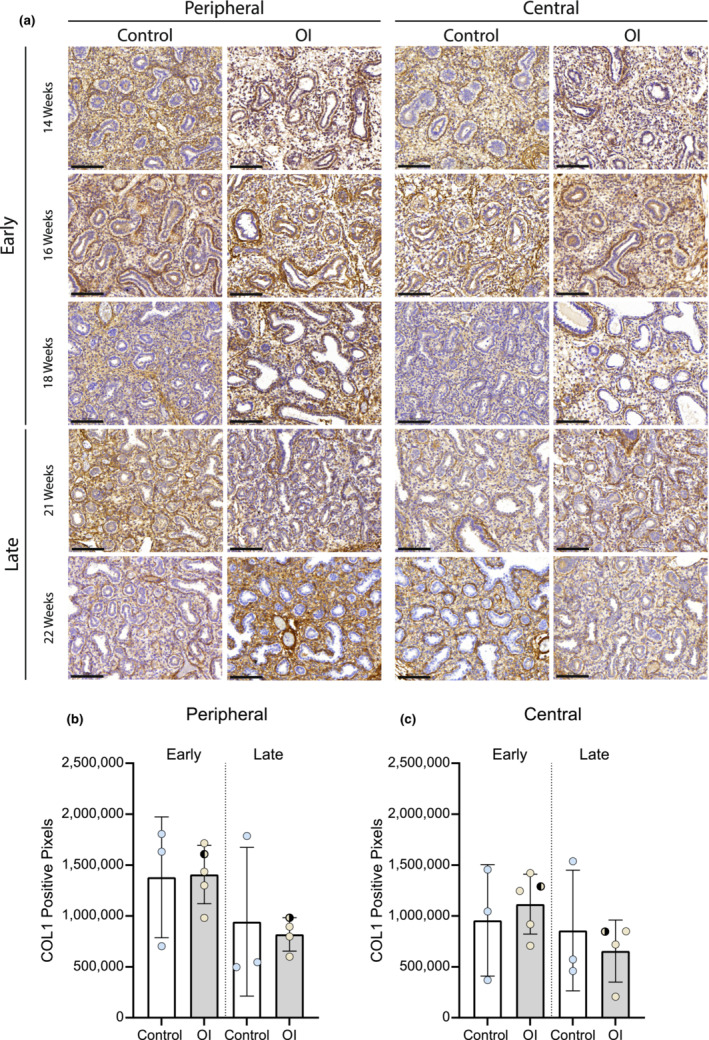
Collagen type 1 expression in OI and control fetuses. Type 1 collagen expression in OI and control fetuses at the indicated gestational ages. Scale bar is 100 μm. 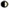: fetus 005 (*PPIB*). 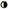: fetus 007 (*COL1A2*). The one‐way ANOVA statistical test was used. Error bars indicate the SD.

**FIGURE 4:**
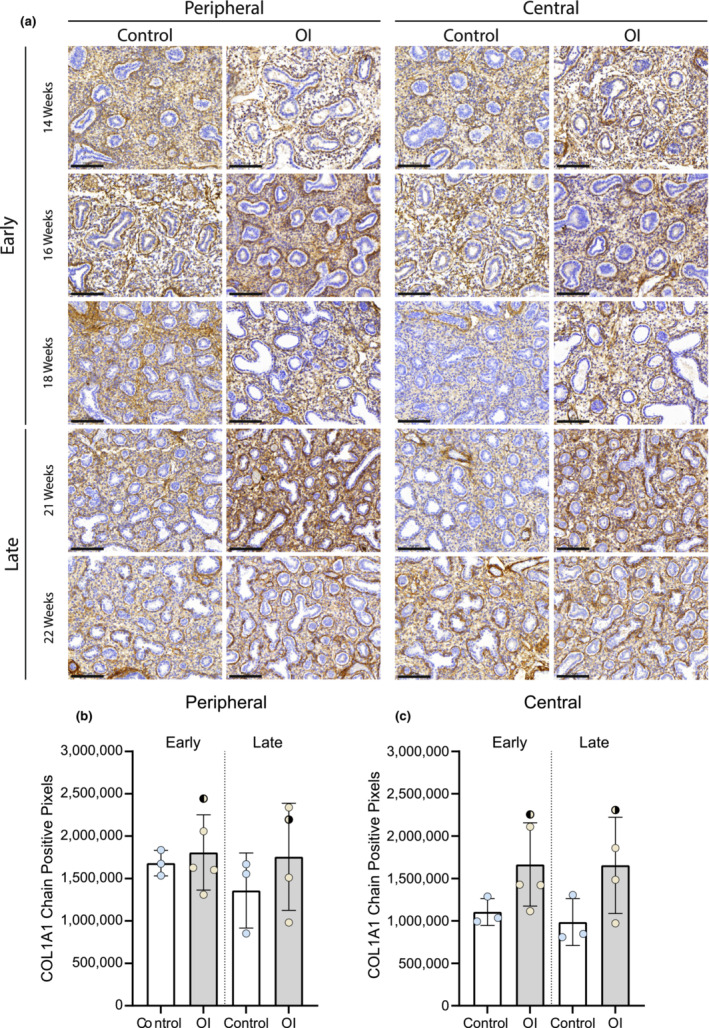
COL1A1 chain expression in OI and control fetuses. Collagen alpha1 chain expression in OI and control fetuses at the indicated gestational ages. Scale bar is 100 μm. 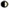: fetus 005 (*PPIB*). 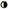: fetus 007 (*COL1A2*). The one‐way ANOVA statistical test was used. Error bars indicate the SD.

**FIGURE 5:**
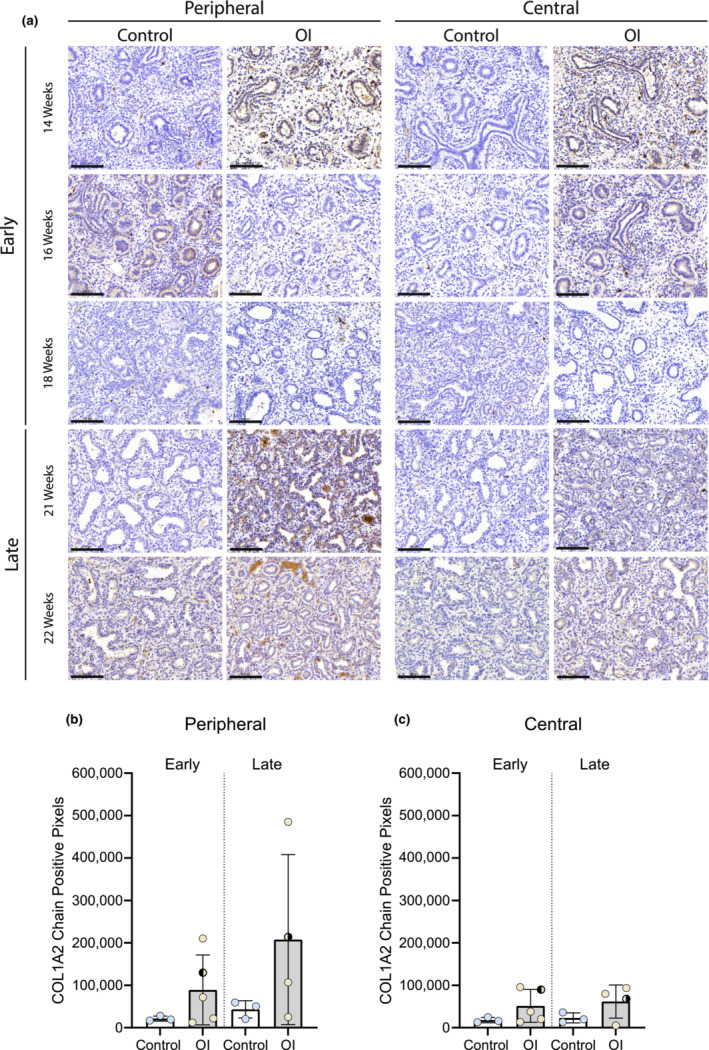
COL1A2 chain expression in OI and control fetuses. Collagen alpha2 chain expression in OI and control fetuses at the indicated gestational ages. Scale bar is 100 μm. 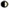: fetus 005 (*PPIB*). 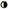: fetus 007 (*COL1A2*). The one‐way ANOVA statistical test was used. Error bars indicate the SD.

**FIGURE 6:**
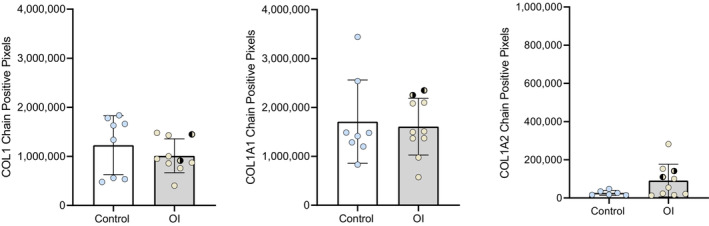
Total positive pixels counting of COL1, COL1A1 chain, and COL1A2 chain. COL1, COL1A1, and COL1A2‐positive pixels in early and late GA in both peripheral and central parenchyma. Unpaired *t*‐test was used. Error bars indicate the SD.

**FIGURE 7:**
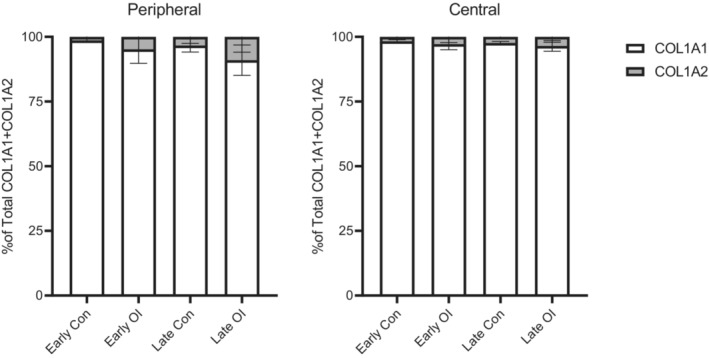
Ratio of pixels counting for the COL1A1 and COL1A2 chains.

Collagen I staining showed no significant differences between the OI and control group (Figure 3). However, analysis of the single collagen chain expression showed differences, although not significant (Figures 4 and 5). The expression of alpha2(I) chains was higher in OI fetuses than in controls, especially in the lung periphery (Figure 5). Although less noticeable, also the expression of alpha1(I) chains appeared to be higher in OI fetuses than in controls, especially in the central part of the lung (Figure 4c). Examining the average number of pixels (central+peripheral and early+late), the expression of collagen I was similar in the OI group compared to controls (Figure 6). The ratio between alpha1(I) chain and alpha2(I) chains pixel counting was lower in the OI group compared to controls, especially in the peripheral part of the lung (Figure 7).

## 4 DISCUSSION

In recent years, awareness on the severity of respiratory disease in OI patients has increased (Storoni et al., 2021). It has been recognized that acute and chronic respiratory problems are responsible for a decreased quality of life, as well as being a major cause of death in OI patients (Folkestad, 2018; Yonko et al., 2020). However, unlike for bone and other connective tissues, the consequences of collagen defects on lung function and development have long been neglected. Scarce information exists on if, and to what extent, abnormal collagen I regulation in the lungs can influence lung development and structure, and whether it can explain the lung hypoplasia that contribute to the high mortality in OI type II patients. This is the first study to examine the intrinsic characteristics of lung parenchyma in OI type II patients, shedding light on both the pneumocyte differentiation and the potential role of collagen as a possible pathophysiological cause of lung hypoplasia in OI type II.

For this purpose, this study aimed at first investigating the differentiation status of type 2 pneumocytes in OI fetal lungs, comparing these to healthy controls of the same GA. Patients were selected from the last part of the pseudoglandular up to the canalicular fetal developmental stage. The use of TTF‐1, which can assess pneumocyte differentiation in surfactant‐producing cells, allowed us to identify the differentiated cells per respiratory duct (Kimura, 1996). Pneumocyte differentiation occurred earlier in OI fetuses than in GA‐matched controls (Figure 2). In OI fetuses, differentiation of the cuboidal epithelium into type 2 pneumocytes occurred as early as 16 weeks GA (pseudoglandular phase). By contrast, in control fetuses the cell differentiation was only visible from 18 weeks GA, consistent with previous reports in literature (Alcorn et al., 1981; Burri, 1984; Davis & Mychaliska, 2013; Kikkawa et al., 1971). A major difference was found in the peripheral part of the lungs in the early group; OI fetuses had a significantly higher percentage of TTF‐1 positivity than the control fetuses (Figure 2b). By contrast, tissue from OI fetuses in the late group exhibited a lower percentage of TTF‐1 positivity compared to control fetuses, both in the peripheral and the central part of the lungs (Figure 2b,c). Interestingly, the pixel count of alpha2(I) chains was higher in OI compared to controls, especially in the peripheral part of the lungs (Figure 5b). In addition, the lowest alpha1(I) to alpha2(I) ratio was found in the peripheral part of the lungs in the OI group. These findings suggest that altered collagen may play a role in the cell differentiation process. In line with this, there has been experimental evidence showing that the presence of collagen is necessary for the development and branching morphogenesis of the embryonic lung (Spooner & Faubion, 1980). Thus, we can assume that type I collagen plays a role biochemically as a regulator of cellular differentiation. Future studies are needed to further clarify if dominant negative alterations in collagen type I may serve as the intrinsic factor of lung hypoplasia, influencing cell differentiation during embryonic lung development.

The other aim of the study was to assess the expression of type I collagen in fetal lungs. All pathogenic collagen type I gene variants which were identified in the OI patients are predicted to have a dominant negative effect resulting in the posttranslational overmodification of collagen affecting fibril assembly (Torre‐Blanco et al., 1992). Similarly, the pathogenic variant in *PPIB* has been also shown to lead to collagen overmodification (van Dijk et al., 2009). We assessed the expression of collagen in the lung parenchyma of type II OI fetuses by means of pixel counting on immunohistochemically stained slides. Although the pixel count of the total type I collagen immunoreactivity showed no significant differences between the two groups, a recognizable pattern was observed for the alpha chains. The ratio of alpha1(I) to alpha2(I) chains was lower in OI than in controls, suggesting that the pathogenic variants in *COL1A1* may result in alpha1(I) chain instability or higher synthesis of alpha2(I) chains. In normal human fibroblasts, concentrations of alpha1(I) and alpha2(I) chains show a ratio of approximately 2:1 (Micha et al., 2020). The concentrations of alpha1(I) and alpha2(I) chains seen in our groups remains not entirely understood. We suppose that this ratio may potentially vary, as a result of collagen instability due to its damaged structure, as a result of pathogenic variants in the collagen type I genes.

Our study is limited by the small number of fetuses, which did not allow us to draw straightforward statistical conclusions. The different mutations of the OI fetuses may also contribute to the variability. Unfortunately, it was not possible to perform staining for the other collagen types as available material was limited. Another limitation of the study is the uncertain accuracy of the immunohistochemical staining for alpha1(I) and alpha2(I), which may not entirely reflect the level of gene expression. However, this is the first study evaluating lung development in type II OI fetuses by also examining the relationship between type I collagen and cell differentiation. Understanding the pathophysiology of lung hypoplasia in patients with OI type II is the basis for future studies and therapeutic proposals.

In conclusion, the development of the lung parenchyma in patients with OI type II is impaired, with early cellular differentiation of the cuboidal epithelium into type 2 pneumocytes. This differentiation occurs at 16 weeks (pseudoglandular phase) in fetuses with type II OI, 2 weeks earlier than in control fetuses in which cell differentiation begins from 18 weeks (canalicular phase). The cause of early cell differentiation is still not entirely clear. We can speculate that the altered collagen may play a role in the cell differentiation process, as demonstrated by the amount of alpha2(I) chains being higher in subjects with OI than in controls and that the ratio of alpha1(I) to alpha2(I) being lower in OI than in controls. The interaction between pneumocytes and the extracellular matrix is not yet fully elucidated, but it is plausible that type I collagen plays an important role as biochemical regulator of cellular differentiation.

## AUTHOR CONTRIBUTIONS

All authors were involved in study conception/design, data analysis, and/or interpretation and in writing/critical review of draft versions of this manuscript. All authors approved the final version for submission and publication. Several authors of this publication are members of the European Reference Network for BONeDiseases ‐ Project ID No 101085766.

## CONFLICT OF INTEREST STATEMENT

The authors declare no conflicts of interest.

## ETHICS STATEMENT

This research study conformed to the Declaration of Helsinki principles and was approved by the Medical Ethics Review Committee (MERC) of the Amsterdam UMC (Amsterdam, The Netherlands).

## Supporting information


Table S1
Table S2

Figure S1

## Data Availability

The original contributions presented in the study are included in the article. Further inquiries can be directed to the corresponding author. Access to de‐identified data or related documents can be requested through the submission of a proposal with a valuable research question, necessary data protection plan, and ethical approvals.
