# Supplementary figures and images for: Altered collagen I and premature pulmonary embryonic differentiation in patients with OI type II
Source: Physiol Rep. 2023 Jul 4;11(13):e15737. doi: 10.14814/phy2.15737 (PMC10318393; doi:10.14814/phy2.15737)

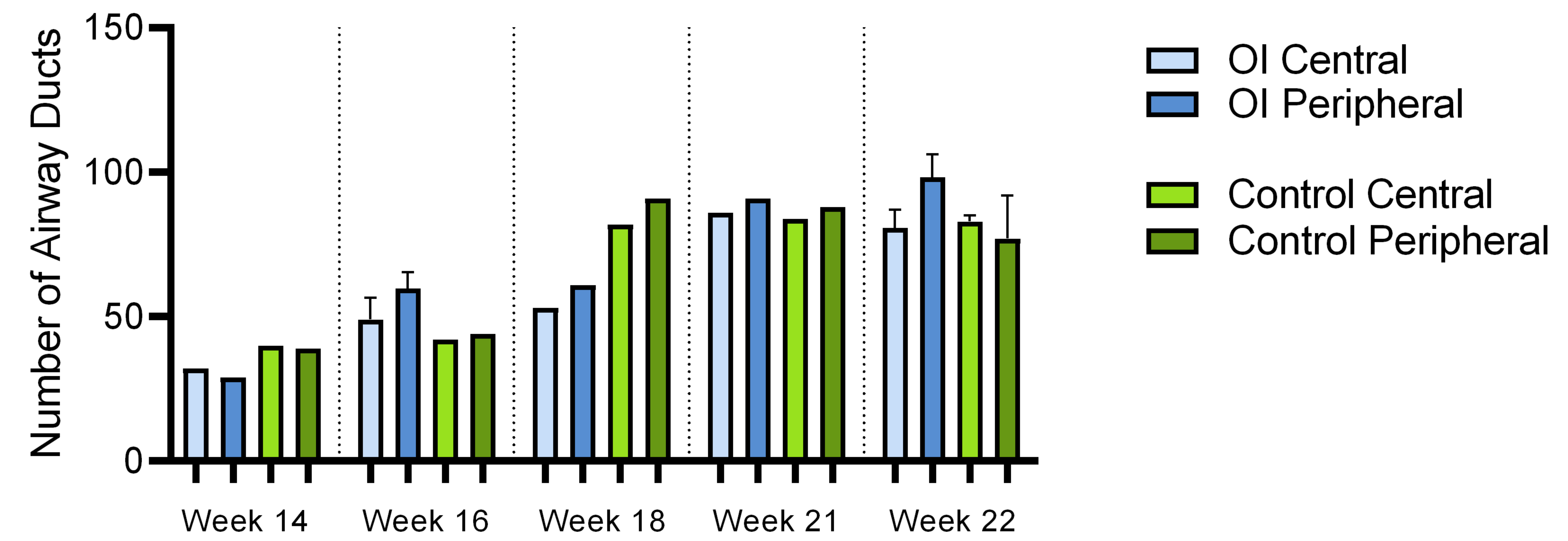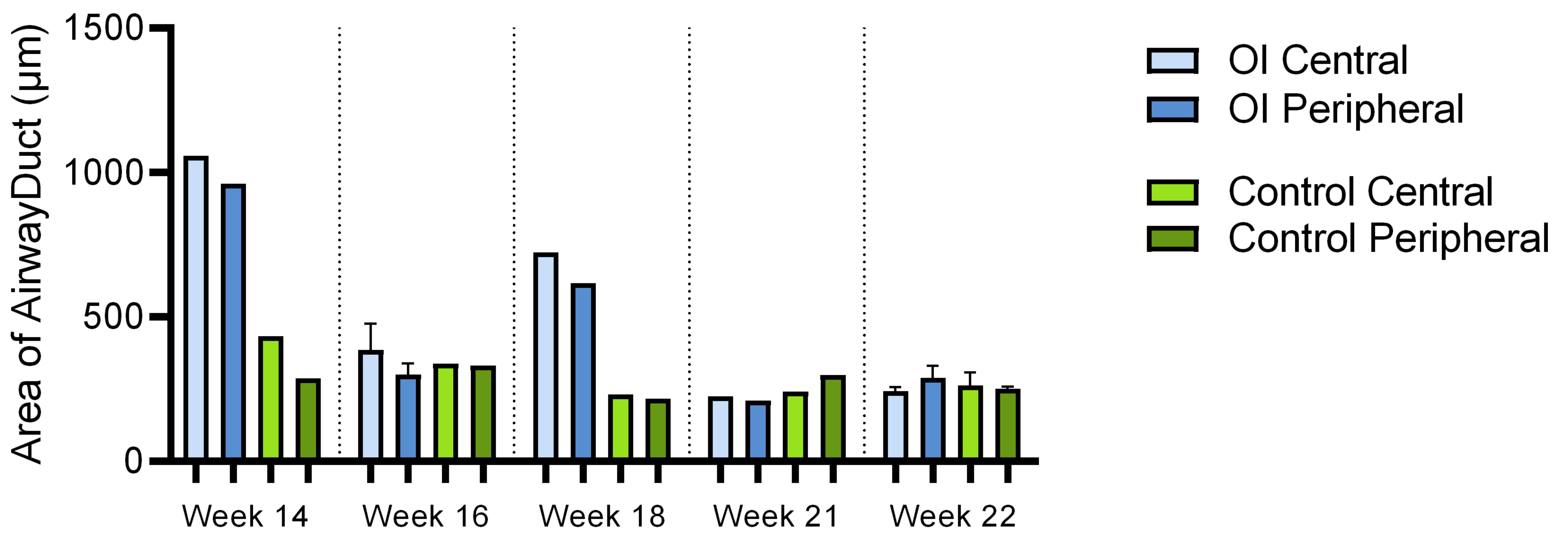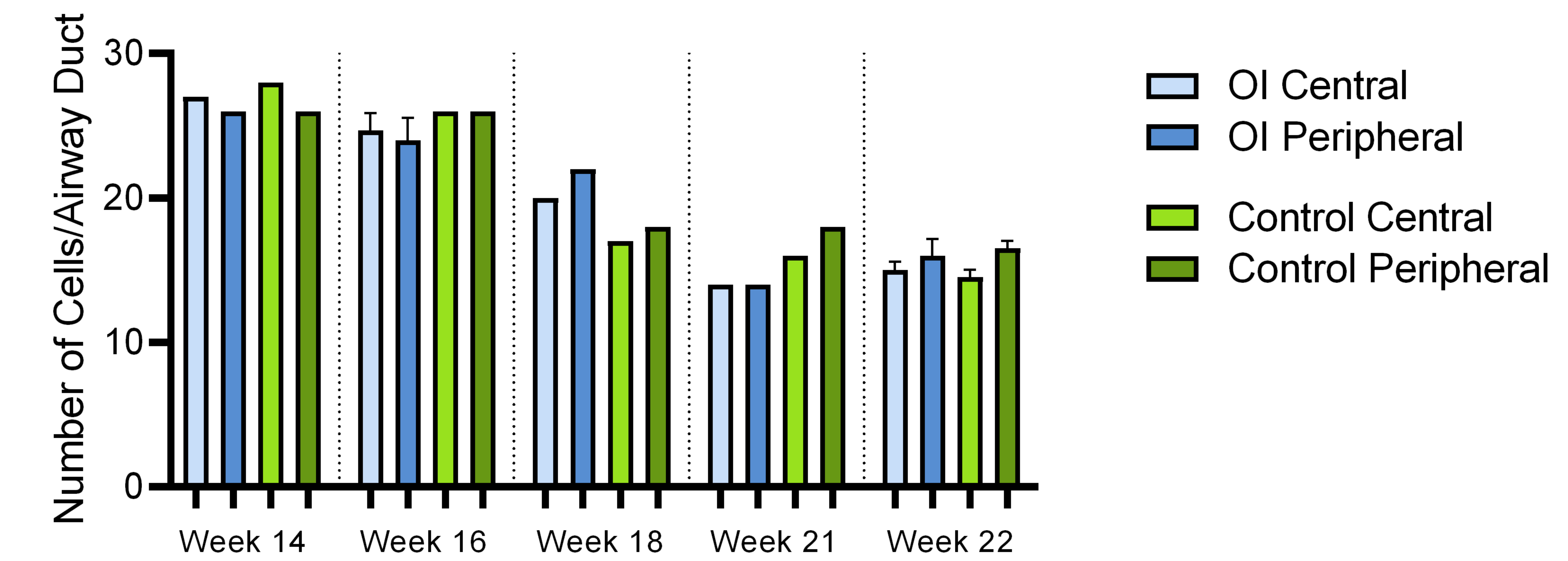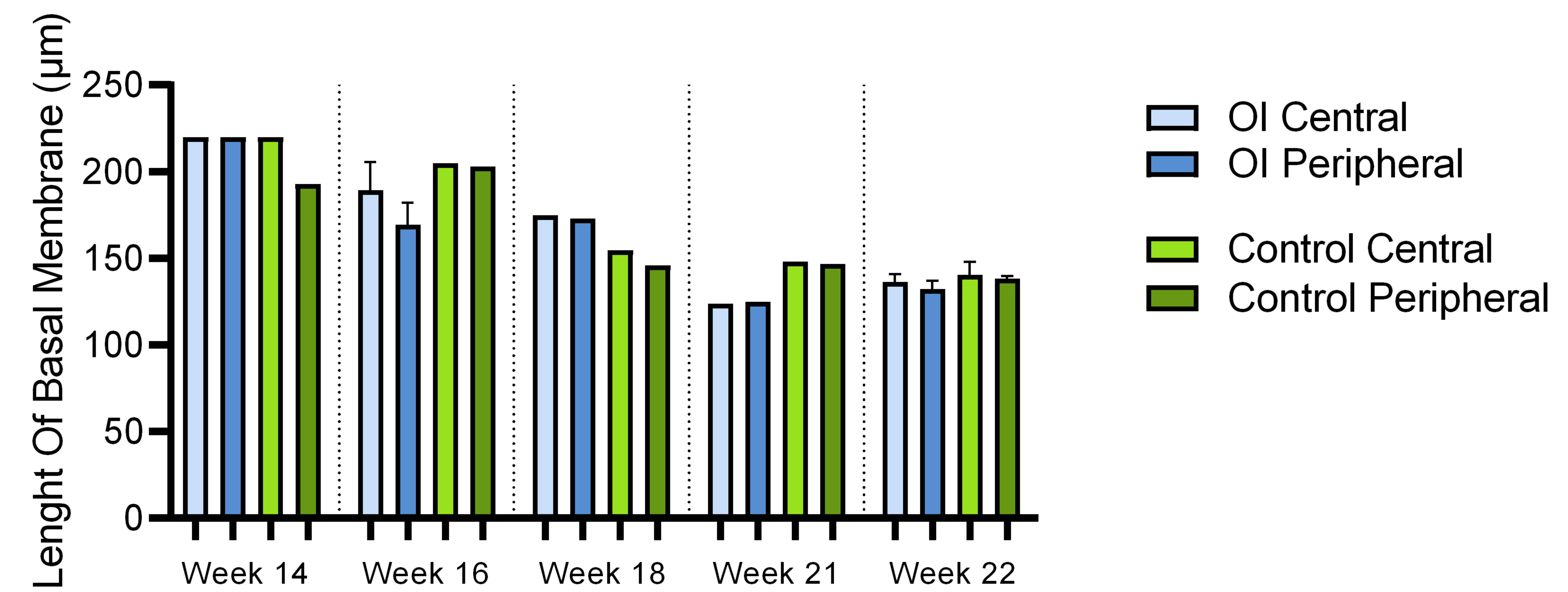

Supplement: Supplementary file 1 — Table S1 Table S2 [file PHY2-11-e15737-s001.pdf]
