# Supplementary material for: Altered collagen I and premature pulmonary embryonic differentiation in patients with OI type II
Source: Physiol Rep. 2023 Jul 4;11(13):e15737. doi: 10.14814/phy2.15737 (PMC10318393; doi:10.14814/phy2.15737)
Supplement: Supplementary file 2 — Figure S1 [file PHY2-11-e15737-s002.docx]

**Table 1S. Airways duct characteristics in OI fetus and control fetus.** Unit of length µm. Unit of area µm^2^.

**Table 2S**. **Ratio of pixels counting for the COL1α1 and COL1α2 chains.**

**Figure 1S. Airways duct characteristics in OI fetus and control fetus on central and peripheral lung regions.** In different developmental stages: number of airway ducts; area of airway ducts, unit of area µm^2^; number of cells per airway duct; length of basal membrane, unit of length µm.
